# Applicability of Laparoscopic Nephrectomy in the Treatment of Multicystic Dysplastic Kidney: Sorting Out Surgical Indication

**DOI:** 10.7759/cureus.2014

**Published:** 2018-01-01

**Authors:** Carlos Augusto F Molina, Jose Bessa Junior, Andrey G Estevanato, Gustavo S Viana, Inalda Facincani, Jose Murillo Bastos Netto, Silvio Tucci Junior

**Affiliations:** 1 Division of Urology, Department of Surgery and Anatomy, Ribeirão Preto Medical School of University of São Paulo (fmrp-Usp); 2 Division of Urology, Department of Surgery, Universidade Estadual De Feira De Santana - UEFS; 3 Division of Urology, Department of Surgery and Anatomy, Ribeirão Preto Medical School of University of São Paulo (fmrp-Ùsp); 4 Division of Pediatric Nephrology, Department of Pediatrics and Childcare, Ribeirão Preto Medical School of University of São Paulo (fmrp-Usp); 5 Division of Urology, Department of Surgery, Federal University of Juiz De Fora (ufjf)and Hospital E Maternidade Therezinha De Jesus of the School of Medical Sciences and Health of Juiz De Fora (hmtj/suprema)

**Keywords:** children, laparoscopy, multicystic displasic kidney, congenital malformations

## Abstract

Introduction

We evaluated the applicability of laparoscopic nephrectomy in the treatment of multicystic dysplastic kidney (MCDK) in children, including procedures performed by resident physicians or trainees in surgical urology.

Methods

We retrospectively evaluated the medical records of 20 children with MCDK who underwent laparoscopic nephrectomy over a six-year period. Data collected included gender, laterality of the affected kidney, age at the time of surgery, the largest diameter of the multicystic kidney and associated urological diagnoses, surgical, and postoperative data. All surgical procedures were performed transperitoneally.

Results

The mean age at the time of surgery was 46 months with a slight predominance of girls. The right side was the more affected side, and the average diameter of the affected kidney at the time of surgical excision was 5.8 cm. Other changes in the urinary tract were found in five children. The mean operative time was 133 minutes. On pathological examination, on average, the pieces measured 4.8 cm and weighed 18.1 g. There were no operative complications. The average length of hospital stay was 37.35 hours.

Conclusion

Laparoscopic nephrectomy was confirmed as an applicable treatment for MCDK. The technique was easy to perform, safe and efficient, even when performed by trainees in pediatric urology.

## Introduction

Multicystic dysplastic kidney (MCDK) is the most common cystic kidney disease in children [1], with a reported incidence of 1 in 4,300 live births [2]. In the 1970s, MCDK was considered as a rare cause of abdominal mass to be treated by nephrectomy [3].

Since then, the use of ultrasonography as a safe prenatal diagnostic method for urinary tract malformations [1] hugely contributed to the increase in the reported number of cases of MCDK, also considering those that do not evolve into the distinct clinical form [3].

Contralateral kidney hypertrophy will be expected, thus considered an indication of renal health; however, it has not been observed in all patients with MCDK. Onal and Kogan showed that on evaluating the contralateral kidney, compensatory hypertrophy was found in 77% patients’ follow-up and they correlated it with involution of the MCDK [4].

To study compensatory hypertrophy in contralateral kidneys of patients with MCDK, Gaither, et al. reviewed 61 MCDK patients with long-term follow-up evaluating predictive factors (including MCDK involution) for it. In their conclusions, they identified a small minority of patients (approximately 10%) who are at higher risk for renal insufficiency, i.e, whose MCDK did not involute or manifest compensatory hypertrophy by age two years [5].

The incidental detection caused an increase in the number of diagnosed cases, as well as better understanding of the natural history of the disease, generating controversy on the need to surgically remove the MCDK [6]. The possibility of hypertension and risk of malignancy in these MCDK cases warrants nephrectomy, advocated by the features and benefits of minimally invasive procedures such as laparoscopy [6]. However, the conservative approach is gaining popularity based mainly on a high rate of spontaneous resolution and insufficient data in the literature about the occurrence of hypertension and cancer in patients with MCDK [6].

Hayes, et al., in 2012, evaluated 323 patients with MCDK for 25 years. After 10 years of follow-up, they reported complete involution in 76% of the kidney units with MCDK smaller than 5 cm, and in 21% of those larger than 5 cm. In the same study, 91% of the kidney units with multicystic dysplasia less than 3 cm disappeared in five years, while none of those measuring more than 7 cm regressed [7].

The possibility of spontaneous resolution and disappearance of the MCDK delayed surgical intervention. Longer follow-up time reflected an increase in the treatment costs of MCDK as noticed by Yamataka, et al. in 2005 [8]. Consequently, surgical treatment was recommended to be performed as early as possible by its advocates [9].

The most appropriate method for the treatment of MCDK, whether medical or surgical, remains unanswered, requiring prospective randomized studies to reach definitive conclusions [10].

In our division, nephrectomy for MCDK is indicated in children with kidney units ≥ 5 cm in size after the first year of age, and for kidney units < 5 cm showing no regression after three subsequent years of outpatient follow-up. Laparoscopy is the first choice to perform nephrectomy in children with MCDK.

This study aims to evaluate the results, safety, and applicability of laparoscopic nephrectomy performed by residents in the treatment of children with MCDK, which, although considered a benign condition, may influence contralateral renal hypertrophy.

## Materials and methods

We retrospectively analyzed the medical records of children with MCDK attending our urology and pediatric nephrology clinics, who underwent laparoscopic nephrectomy between 2008 and 2013.

The study included a total of 20 children with MCDK consecutively evaluated at a pediatric urology clinic. The study was approved by the Institutional Research Ethics Committee (number 5865/2014), and the need for informed consent was waived. The laparoscopic approach was proposed to parents, explaining the risks and benefits involved, and performed after receiving their approval. 

The following data were collected:

·  Patient data: gender, laterality of the affected kidney, age at the time of surgery, the greatest diameter of the multicystic kidney on ultrasonography, and other associated urological diagnoses.

·  Surgical data: number of ports used for trocar introduction, operative time, and intraoperative complications.

·  Postoperative data: length of hospital stay, largest diameter and weight of the kidney at biopsy, and complications during hospitalization and follow-up. The length of hospital stay was defined as the time from the end of surgery to discharge.

All procedures were performed transperitoneally. The staff introduced an orogastric tube under anesthesia to aspirate the stomach contents in all patients. The patients were probed with Foley’s catheter and then positioned in the 45° modified flank position. The first trocar was inserted through the umbilicus, using the Hasson’s technique, for placement of the laparoscopic camera and peritoneal insufflation (between 10-12 mmHg). For a right nephrectomy, two 5 mm hits were positioned (in the midline near the xiphoid process and the right mammary line at the level of the umbilicus). A 10 mm access was created in the midline between the xiphoid process and the umbilicus. For a left nephrectomy, a 5 mm access was created in the midline between the xiphoid process and the umbilicus and another 10 mm access in the mammary line at the level of the umbilicus. The surgery began with folding the ipsilateral colon down the right side and removing the liver superiorly to expose the kidney area using the closer access to the xiphoid process. The kidney hilum was dissected and connected with titanium clips or "hemolock" and transected. After the release of the whole kidney, the ureter was sectioned with monopolar electrocautery, and larger cysts were aspirated to facilitate removal of the specimen through the umbilical access.

No additional access was necessary. Adequate hemostasis was achieved by electrocautery. Hence, no surgical drain was left in the abdominal cavity. The 10 mm access through the aponeurosis was sutured with Vicryl 4-0. The bladder catheter was removed on the next day, and the patients were discharged home after commencing oral feeding and achieving adequate pain control. The first postoperative evaluation was performed one month after surgery in the outpatient clinics.

## Results

Twenty children with MCDK admitted for laparoscopic nephrectomy in our hospital between 2008 and 2013 were included. All surgeries were performed by the institution’s staff and urology residents who were in their last year of training. Among the patients, there was a slight predominance of girls and the right side. The kidney diameter at ultrasonography did not differ between the first year and at the surgical indication. The baselines are summarized in Table 1.

**Table 1:** Characteristics of the patients (N = 20) MCDK - multicystic dysplastic kidney, VUR - vesicoureteral reflux.

Age (months)	46.5± 4.23
Gender	
Male	9 (45%)
Female	11 (55%)
MCDK Laterality	
Right	12 (60%)
Left	8 (40%)
Kidney diameter - DRDM (cm)	
1^st^ year of life	5,96
Surgical indication	5,85
Associated urological diagnoses	
Ipsilateral/contralateral VUR	2 (10%)
Ipsilateral ureterocele	2 (10%)
Ipsilateralpelvic kidney	1 (5%)

The mean operative time was 133.1±14.7 minutes. For right-sided nephrectomy, four ports were utilized, and for left-sided nephrectomy, we used three except in one case. On pathological examination, all kidneys were compatible with the clinical diagnosis of dysplastic multicystic kidney disease. There were no significative operative complications, and all subjects had an uneventful evolution despite one episode of bronchospasm, with good response to clinical measures. The length of hospitalization was 37.35 ± 4.11 hours. On pathological examination, the operative specimens measured 4.8±1.1 cm and weighed 18.1 ± 2.07 g.

At the first month after surgery, one child reported secondary nocturnal enuresis that disappeared after three months. The follow-up period ranged from two to seven months.

## Discussion

MCDK is the most common cystic kidney disease diagnosed nowadays using the gestational/perinatal ultrasonography (US) and can be managed by follow-up or surgically (removal of the affected kidney). Hypertension, urinary tract infection (UTI), and Wilms tumor are complications of MCDK. The fact that the absolute risk is unknown is a challenge and makes the follow-up of patients necessary and essential [9,11].

MCDK is considered a benign condition, although a slight chance of development of hypertension, urinary infection, and cancer in these patients have been reported. Another concern that has been brought to question recently is that an involutional MCDK would be able to influence contralateral compensatory kidney hypertrophy [5]. Furthermore, the compensatory hypertrophy is directly linked to renal health, which is strictly correlated with patient morbidity and mortality during adulthood.

Clinical follow-up comprises US, urinalysis, and monitoring of systemic blood pressure level, with questions about the frequency and duration of this control [10]. On the other hand, surgical treatment ends the follow-up of these patients, bringing down morbidity and cost. This raises the question about the best surgical approach, open or laparoscopic.

Minimally invasive surgery has revolutionized pediatric surgical urology in many ways. Today, performing laparoscopic nephrectomy is a well established, safe, and technically efficient practice with less morbidity. Therefore, it stands as a good option for surgical treatment of MCDK [10,12]. The overall complication rate of laparoscopic nephrectomy is around 2.7% and tends to decrease with more experienced surgical teams [11].

In 2005, Yamataka, et al. conducted a study to compare the cost of clinical monitoring, including the necessary examinations, with the cost of surgical treatment and hospitalization. He concluded that for MCDK that spontaneously regress before five years of follow-up, the cost was lower compared to that of the initial surgical treatment [8].

Comparing open to laparoscopic surgery in the pediatric age group, Cervillione, et al., in 2007, showed that laparoscopy was 54% less expensive, considering the time of hospitalization and use of disposable material [13]. They also reported an insignificant difference in the operative time for both approaches. From the parents’ point of view, laparoscopy is aesthetically much better than open surgery [10]. Thus, considering the better feasibility and safety, laparoscopic nephrectomy for MCDK should replace conventional open nephrectomy [14].

Despite reaching a satisfying answer for the best surgical approach for nephrectomy, the question about the best management for MCDK, whether medical or surgical, remains unanswered [7].

Eickmeyer, et al., 2014, analyzed a sample of 301 multicystic kidney units and identified the kidney size as the only significant predictor of the possibility of spontaneous kidney involution by multivariate and bivariate analyses. MCDK less than 5 cm in the largest diameter disappeared at an average age of 2.7 years, while units more than 5 cm in the largest diameter had a mean involution age of 11 years. The fact that a total of 53.5% of children showed the spontaneous disappearance of the kidney on ultrasonography at the age of 10 years without developing malignancy, justifies non-surgical management and follow-up with imaging studies] [15]. As previously discussed, the non-surgical approach often requires long-term follow-up [1], with imaging, laboratory tests, and medical appointments, which increases the cost of treatment. In addition, the risk of infection, hypertension, and cancer is higher especially if the patient is lost to follow-up [14]. Persistence of the MCDK indicates the failure in non-surgical treatment, which is an indication for surgical treatment. This will consequently increase the final cost.

In updating the management of MCDK, Cardona-Grau and Kogan [16], 2015, reported that nephrectomy should no longer have a role in the treatment of MCDK, motivated by the knowledge of its natural history and the reports of involution of MCDK presented by Eickmeyer, et al. [15]. However, despite stressing the importance of follow-up and the need to reduce the number of exams, they did not mention the duration, frequency, or cost of follow-up [16-18].

Greater tolerance to surgical treatment is demonstrated by less analgesic requirement postoperatively, lower complication rate, early ambulation, and rare adverse effects [19]. Questioning parents about the preferable technique for nephrectomy, open vs. laparoscopic, Yamataka, et al., in 2005, found a preference for laparoscopy over open surgery because of shorter hospitalization, lower degree of surgical stress, and better appearance of the scar over time (post-cosmetic surgery) [8].

Knowing that laparoscopic nephrectomy is considered and accepted as a minimally invasive, safe, and efficient procedure for the treatment of MCDK, it should be offered as an appropriate treatment option to families of children with unilateral MCDK [8,10].

In an attempt to establish a flowchart for the management of MCDK that answers the existing questions, aligns cost and morbidity, and optimizes the benefits of clinical presentation and surgical approach, our division established laparoscopy as the technique of choice for nephrectomy in children with MCDK [17]. It is indicated for the dysplastic kidney with size > 5 cm after the first year of life, or < 5 cm with no signs of regression in three subsequent years of clinical follow-up.

In reviewing the medical records of our patients over six years, it was found that their characteristics were similar to those described in the literature and that the results of surgery and postoperative follow-up were excellent. There was no difficulty in the application of the laparoscopic technique or operative complication necessitating the conversion to open surgery. The operative time was reasonable, approximately two hours, considering that the procedure was performed, in part or in full, by urology residents.

Arap, et al. in 2013, described a series of 90 consecutive laparoscopic pyeloplasties performed by residents over a period of six years with a low complication rate (4.4% in total). They considered the procedure to be feasible and with high success rate even when done by medical trainees [19]. The complication rate in our study was in line with that of Arap’s study. There was only one episode of bronchospasm, which did not affect the length of hospital stay, evidenced by the proximity between its average and median values and its absolute value of 41 hours. Another unique complication was secondary nocturnal enuresis as an isolated symptom that resolved during the first year of outpatient follow-up. These complications should be regarded as secondary to surgical management rather than laparoscopy per se.

Surgery was postponed upon the request of the parents of some children with an indication for surgery in the first year of life, the kidney size of the dysplastic unit being > 5 cm. The parents were not comfortable with the idea of surgery, fueled by the possibility of involution of the MCDK in the future. These children were followed up clinically and operated upon in the subsequent three years.

The incidence of contralateral compensatory kidney hypertrophy between postponed surgery patients and not postponed was not analyzed, and it is thereby a target for future study.

## Conclusions

In our experience, laparoscopic nephrectomy was confirmed as an applicable, easy-to-perform, safe, and efficient surgical technique of choice when surgical treatment is indicated for MCDK, even when performed by pediatric urology trainees. The harms of laparoscopic nephrectomy are very low, and removing the kidney after an initial period of observation might obviate the need for imaging tests and strict follow-up. To sum up, we firmly believe that laparoscopic nephrectomy is the best treatment for MCDK measuring more than 5 cm at the end of the first year of life. Further studies regarding the influence of MCDK on contralateral compensatory kidney hypertrophy will enlarge and strengthen the surgical approach.
